# Alcohol Abuse as a Risk Factor for and Consequence of Child Abuse

**Published:** 2001

**Authors:** Cathy Spatz Widom, Susanne Hiller-Sturmhöfel

**Affiliations:** Cathy Spatz Widom, Ph.D., is a professor in the Department of Psychiatry, New Jersey Medical School, Newark, New Jersey. Susanne Hiller-Sturmhofel Ph.D., is a science editor of Alcohol Research & Health

**Keywords:** child abuse, AOD (alcohol or other drug) abuse, risk factors, family AODU (AOD use, abuse, and dependence) history, family dysfunction, marital conflict, sexual abuse, coping, antisocial behavior, posttraumatic stress disorder

## Abstract

The relationship between child abuse and the use or abuse of alcohol has two aspects. First, some findings have indicated that parental alcohol abuse may be associated with the physical or sexual abuse of children. Research findings in this area remain inconsistent, however. Second, the experience of being abused as a child may increase a person’s risk for alcohol-related problems as an adult. This relationship has best been demonstrated in women who had been victims of childhood abuse. Several factors most likely contribute to or influence this relationship, including coping skills; antisocial behavior; and psychological problems, such as posttraumatic stress disorder.

According to research estimates, each year more than 1 million children in the United States experience some form of abuse or neglect (Widom 1993). Child abuse is one of the many types of violence associated with alcohol use and abuse, either as a consequence or as a causative factor. For example, parental alcohol abuse may contribute to the abusive treatment of children. Furthermore, people who have been abused as children may be at increased risk for developing alcohol abuse as adults.

Child abuse manifests in various forms, including physical abuse, sexual abuse, neglect, and emotional or psychological abuse (Widom 1989). Physical abuse refers to all types of maltreatment that result in physical injuries, such as bruises, welts, burns, abrasions, lacerations, cuts, or fractures. Sexual abuse also can encompass a variety of abusive behaviors, ranging from fondling or touching to sodomy, incest, or rape. Neglect is defined as any situation in which a child receives no care by a parent or other primary caregiver or receives care that is below acceptable community or professional standards (e.g., fails to provide adequate food, clothing, shelter, or medical attention). Emotional and psychological abuse, which may occur in conjunction with the other types of abuse previously mentioned, also can have profound long-term consequences for the child. Because this last type of abuse is difficult to define and identify, however, most research does not explicitly include emotional abuse in child abuse studies. Furthermore, few studies have investigated specifically the relationship between child neglect and alcohol use.

The first part of this article reviews studies assessing the alcohol-related and non-alcohol-related factors that might contribute to parental child abuse, although these studies have produced inconsistent findings.

The second part of this article includes more conclusive research findings concerning the relationship between childhood victimization, particularly childhood abuse and neglect among women, and subsequent adult alcohol abuse. Within this discussion, the article explores how future research may identify further characteristics that could increase a person’s risk for developing alcohol abuse as a consequence of childhood victimization.

## Factors That Contribute to Parental Child Abuse

Researchers have suggested that numerous factors play a role in parental child abuse. Some factors directly relate to parental alcohol abuse, whereas other factors do not—or only do indirectly.

### Physical Abuse

Although many people might intuitively assume that parental alcohol use and abuse contributes to child abuse, research in this area frequently has produced inconsistent results (Widom 1993). For example, some early studies on the relationship between parental alcohol abuse and parental perpetration of physical child abuse found only modest associations (see Miller et al. 1997). Other studies detected either no associations or associations limited to certain subgroups of alcohol-using parents (see Miller et al. 1997). These studies, however, frequently suffered from methodological limitations.

Despite improvements in methodology, more recent studies also have found inconsistent results regarding the association between parental alcohol use and child abuse. For example, a study among college students evaluated the participants’ recollection of childhood physical, sexual, or emotional abuse and of parental alcoholism, but found no significant relationship between parental alcohol use and the various types of child abuse (Harter and Taylor 2000). Conversely, other retrospective studies determined that a parent’s alcohol problems were related to that parent’s violence against the child (see Miller et al. 1997).

Other studies have determined the child abuse potential (i.e., the types of discipline imposed) of parents with and without histories of alcohol and other drug (AOD) abuse. Ammerman and colleagues (1999) found that parents with histories of AOD abuse had higher child abuse potential than did parents without such histories.^1^ In another study, mothers with histories of alcohol problems were more likely to use harsh punishment on their children compared with women without such histories (see Miller et al. 1997). These results provide some support for the hypothesis that parental alcohol abuse may be associated with physical child abuse. However, further research is needed before firm conclusions can be drawn about the extent and nature of the connection between parental alcohol abuse and subsequent child abuse.

On the assumption that a relationship does exist between parental alcohol problems and child abuse, researchers have begun to speculate about some of the possible mechanisms linking these problems. For example, Miller and colleagues (1997) have suggested the following three possible mechanisms:

- *The cognitive disorganization* hypothesis posits that alcohol abuse increases the likelihood of violence, because it interferes with communication among family members and results in misinterpretation of social cues, overestimation of perceived threats, and underestimation of the consequences of violence.
- The *deviance disavowal* hypothesis suggests that the perpetrator attributes the violence to his or her alcohol abuse and thus avoids or minimizes personal responsibility for the violent behavior.
- The *disinhibition hypothesis* proposes that alcohol’s pharmacological actions on the brain interfere with the actions of those brain centers that control (i.e., inhibit) socially unacceptable behaviors.

### Sexual Abuse

The relationship between parental alcohol abuse and childhood sexual abuse (CSA) may be even more complex, because the perpetrator of the abuse may be the alcohol-abusing parent or another person.^2^ For example, several studies found that CSA experiences for both men and women were associated with family histories of alcoholism (Miller et al. 1997). Similarly, Vogeltanz and colleagues (1999) identified parental drinking as a risk factor for CSA. Concurrently, most victims were abused by either another family member or by a stranger (Miller et al. 1997), suggesting that parental alcohol abuse may leave children more vulnerable to sexual abuse by others.

Fleming and colleagues (1997) have supported the aforementioned hypothesis and have found that several factors are associated with a girl’s risk of being sexually abused, such as experiencing physical abuse, having a mother who was mentally ill, being socially isolated, and not having a person in which to confide. Furthermore, whereas an alcoholic father was a risk factor for CSA by a family member, an alcoholic mother was a risk factor for CSA by a person outside the family.

The mechanisms underlying this association between parental alcohol abuse and CSA remain unclear. Possibly, parental alcohol abuse increases children’s vulnerability to CSA by interfering with the parents’ ability to provide a supportive, nurturing, and protective environment (Miller et al. 1997). For example, an alcohol-abusing parent might be less available to protect a child from extrafamilial CSA than a non-alcohol-abusing parent.

### Socioeconomic Status (SES)

Several studies have identified low SES as a factor contributing to child maltreatment (Coulton et al. 1999; Korbin 1998; Drake and Pandey 1996). In addition, a low SES may both result from and contribute to alcohol abuse and dependence. Accordingly, parental alcohol abuse may act together with low SES to contribute to child abuse.

### Marital or Relationship Stress

Research shows that a stressful relationship between parents can markedly increase the risk of child abuse (see Miller et al. 1997; Fleming et al. 1997). For example, when such stress manifests itself in the form of one spouse physically abusing the other, the child also is likely to experience such physical abuse (Ross 1996). Alcohol abuse by one or both spouses can lead to marital stress, including spouse abuse. This observation reinforces the notion that parental alcohol abuse potentially acts through several mechanisms to increase the risk of child abuse.

### Parental History of Abuse

Some studies have suggested that a parent who experienced abuse as a child is more likely to be abusive toward his or her own children, although this connection has not been thoroughly confirmed by research. For example, based on a literature review, Kaufman and Zigler (1987) have estimated that the rate of inter-generational transmission of abuse is approximately 30 percent. This means that one-third of persons who were abused or neglected in childhood will abuse their own children, whereas the majority (i.e., two-thirds) of persons who experienced child abuse will not abuse their own children (Widom 1989). Other analyses have suggested that mothers who have a history of being physically or sexually abused may have difficulty protecting their children from abuse by a spouse or other person (see Miller et al. 1997). Thus, parental abuse history may affect children’s risk of being abused.

## Child Abuse as a Risk Factor for Later Alcohol Abuse

Numerous investigators have analyzed the relationship between childhood physical and sexual abuse and the development of adult alcohol problems. Most of these studies have been conducted retrospectively—that is, adolescent or adult study participants with or without alcohol problems were asked about their childhood experiences of abuse (e.g., Miller et al. 1993; Wilsnack et al. 1997). Fewer studies have been conducted prospectively—that is, have followed abused children through adulthood to determine whether they developed alcohol-related problems (e.g., Ireland and Widom 1994; Widom et al. 1995). Studies also have differed with respect to their participants. Some studies recruited people undergoing treatment for alcoholism or other psychiatric disorders (e.g., Miller et al. 1993), whereas other studies have used general population samples (e.g., Wilsnack et al. 1997). These differences likely influenced the applicability of the study results to the wider population, because not all victims of child abuse seek treatment as adults, and people who do seek treatment may have higher rates of alcohol abuse than people who do not (Widom et al. 1995).

Few studies have investigated the relationship between childhood victimization and later alcohol use in men. In a prospective study, Ireland and Widom (1994) followed 908 children with court-documented abuse or neglect histories and a control group of 667 matched children without such histories. The investigators analyzed whether childhood victimization was associated with an increased risk of AOD-related arrests as juveniles or adults. This analysis found that for male subjects, a history of childhood abuse did not significantly predict AOD arrests. In contrast, such an abuse history significantly predicted adult (but not juvenile) AOD arrests among female subjects. A followup study of the same sample also concluded that no relationship existed between childhood victimization and subsequent alcohol abuse in men, but found a significant increase in risk for women (Widom et al. 1995). An earlier prospective study also detected no increased risk of adult alcohol abuse in physically abused males (see Langeland and Hartgers 1998). Finally, retrospective studies found that the rates of childhood sexual or physical abuse among male alcoholics are similar to or somewhat higher than the rates found in the general population (see Langeland and Hartgers 1998). Overall, insufficient information exists from which to draw firm conclusions about the relationship between childhood victimization and adult alcohol abuse in men (Langeland and Hartgers 1998).

Most studies on the correlation between childhood victimization and adult alcohol abuse have been conducted in women. These analyses have used a variety of samples, including women in the general population, women with court-documented histories of childhood abuse or neglect, and women undergoing alcoholism treatment. With a few exceptions (Widom et al. 1995), most of these studies have focused on CSA.

Wilsnack and colleagues (1997) investigated the relationship between CSA and adult drinking behavior in 1,099 women who participated in a 10-year national survey on women’s drinking. In this study, the investigators assessed CSA retrospectively through self-reports by the participants.^3^ Those women who had experienced CSA were significantly more likely than other women to report one or more of the following alcohol-related behaviors and problems:

- Alcohol consumption in the 30 days before the survey interview
- Intoxication in the year before the survey interview
- One or more alcohol-related problems (e.g., fights with family members, work problems, home accidents, and problems with children) in the year before the interview
- One or more symptoms of alcohol dependence (e.g., memory lapses while drinking, morning drinking, and inability to stop or reduce drinking over time) in the year before the interview.

In another community-based study, Fleming and colleagues (1998) compared the prevalence of CSA among alcohol-abusing women (as identified through their responses to the Alcohol Use Disorders Identification Test [AUDIT]) and non-alcohol-abusing women. The study found that CSA by itself did not significantly predict alcohol abuse. When considered together with other factors in a woman’s family background (e.g., having a mother perceived as cold or uncaring or having an alcoholic partner), however, a history of CSA became a significant predictor of adult alcohol abuse. These findings indicate that no simple relationship exists between CSA and adult drinking behavior and that numerous other factors in a woman’s life influence this relationship.

As mentioned earlier, Widom and colleagues (1995) followed into young adulthood both a large group of people who had been abused or neglected in childhood and a matched control group. The investigators compared the levels of alcohol abuse and dependence in both groups. The study found that for women, a history of childhood neglect (but not abuse) significantly predicted the number of alcohol-related symptoms experienced during adulthood, independent of parental AOD problems, childhood poverty, race, and age. However, neither abuse nor neglect predicted the clinical diagnoses of alcohol abuse or dependence. Possibly, however, such relationships were not detected, because the courts’ intervention in these cases lessened the effect of the abuse on the children and improved the children’s long-term outcomes.

Finally, as mentioned earlier, abused and neglected males in the same study had no increased risk of adult alcohol problems compared with control males. The researchers suggest that the gender difference may result in part from differences in how men and women respond to childhood victimization. For example, one common theory is that men may be more likely to express themselves through externalizing behavior (e.g., aggression). Conversely, women may be more prone to internalizing pain and suffering, which then may lead to self-destructive behaviors, including alcohol abuse (Widom et al. 1995). Another possibility is that the men in the overall sample were at high risk for alcohol abuse (approximately two-thirds of the men in the sample met DSM–III–R^4^ criteria for alcohol abuse and/or dependence diagnosis) for a variety of reasons and that child abuse and neglect may not have been an independent risk factor for subsequent alcohol problems in these men.

Other researchers investigated the prevalence of CSA and other forms of childhood victimization among women undergoing alcoholism treatment and various control groups (Miller et al. 1993). In that study, women receiving alcoholism treatment were significantly more likely to report CSA as well as father-to-daughter verbal aggression and physical violence than women in the general population, women attending classes for first-time drunk-driving offenders, or women receiving treatment for other mental health problems (Miller et al. 1993). This relationship between CSA and alcohol abuse was independent of parental alcohol problems, race, and the number of changes in childhood family structure. Further analyses using the same samples found that women who reported father-to-daughter verbal aggression and violence during childhood were more likely than other women to have low self-esteem (Downs and Miller 1998), suggesting that low self-esteem may play a role in the link between childhood victimization and adult alcohol abuse.

In summary, various studies on the relationship between childhood victimization and adult alcohol-use behaviors have yielded inconsistent results, although several studies have indicated that particularly among women, childhood abuse and neglect may increase the risk for adult alcohol problems. Additional research is needed to clarify this relationship and identify factors that may influence it. Some of those factors are described in the following section.

### Factors Influencing the Relationship Between Childhood Abuse and Neglect and Adult Alcohol Abuse

Researchers have proposed several hypotheses as to why victims of child abuse and neglect may be at increased risk for alcohol abuse during adulthood. Thus, alcohol may serve as the following (see Widom et al. 1995):

- A mechanism to cope with or escape from the trauma of childhood victimization and the related depression
- A way to reduce feelings of isolation and loneliness
- Self-medication in an attempt to gain control over the experience
- A way to improve self-esteem
- A form of self-destructive behavior.

Accordingly, factors such as poor coping skills, antisocial behavior, and abuse-related posttraumatic stress disorder (PTSD) may help mediate the relationship between childhood victimization and adult alcohol problems.

#### Coping

Researchers have suggested that for some victims of childhood abuse, alcohol may serve as a coping mechanism to deal with the trauma associated with the abuse and its consequences (Miller et al. 1997). For example, childhood victimization frequently results in depression. People who lack the proper coping mechanisms (e.g., seeking help from others) to deal with their experiences of childhood victimization and the resulting depression may use alcohol to make themselves feel better. Because alcohol merely covers, rather than cures, the problem, the need for alcohol may persist or even increase over time, increasing the risk of developing alcohol abuse or dependence. This mechanism may be more common in women than in men, because in men depression in most cases appears to be a consequence of rather than a contributing factor to alcohol abuse (see Miller et al. 1997).

Schuck and Widom (in press) also examined the role of coping skills and other behavioral and psychological factors (i.e., depression, isolation and loneliness, feelings of worthlessness, and low self-esteem) in the relationship between childhood abuse and neglect and adult alcohol problems. The study included 582 women with court-documented childhood abuse and neglect. Of the factors studied, only alcohol use as a coping mechanism served as a mediator between child abuse and neglect and subsequent alcohol problems. Thus, child abuse and neglect significantly increased the use of alcohol or other drugs to cope, which, in turn, significantly increased the number of alcohol problems. For the relationship between childhood neglect (but not abuse) and subsequent alcohol problems, depression also was a mediator.

#### Antisocial Behavior

Several studies among children and adolescents who had experienced child abuse found that externalizing and antisocial behaviors (e.g., aggression, violence, hyperactivity, and delinquency) can be a consequence of childhood victimization (see Miller et al. 1997; Widom 1989, 1997). In turn, persistent externalizing and antisocial behaviors are strong predictors of AOD use. To explain this observation, researchers have speculated that children or adolescents exhibiting such oppositional and delinquent behaviors may frequently become involved in deviant peer groups that also promote AOD use. The temporal sequence of these hypothesized relationships needs further examination, however.

The relationship between childhood victimization, antisocial behavior, and adult alcohol problems may apply particularly to victims of child abuse with family histories of alcoholism. Research shows that children of alcoholic parents are at increased risk for alcohol problems themselves (Kendler and Prescott 1997) and that in many cases, the risk is mediated by genetic rather than environmental influences (Prescott et al. 1999).

Researchers have identified several types of alcoholism, one of which is characterized by high levels of antisocial behavior that frequently begins manifesting during adolescence. Children whose parents have this type of alcoholism may be particularly likely to be abused during childhood. In addition, those children are at increased risk for antisocial behavior and subsequent alcohol problems themselves, both because of a genetic predisposition and because of the experience of child abuse. Therefore, future studies of the link between child abuse and later alcohol abuse need to address the potentially confounding effects of a genetic predisposition for alcohol problems (Widom 1993).

#### PTSD

PTSD is caused by a person’s experience of an extremely stressful situation, such as threatened or actual violence toward the person or toward someone close to that person. Symptoms of PTSD include persistent recollections (e.g., dreams) of the trauma, avoidance of any stimuli (e.g., places or people) associated with the trauma, and persistent symptoms of increased arousal (e.g., hypervigilance). PTSD is a relatively common consequence of physical or sexual child abuse (Miller et al. 1997; Widom 1999), and researchers have begun to investigate a possible link between child abuse, PTSD, and adult alcohol problems, particularly among women who previously had experienced CSA. For example, one study found that the prevalence of two or more alcohol problems was significantly higher among women who had been victimized and experienced PTSD symptoms than among women who had not been victimized or who had been victimized but did not experience PTSD symptoms (see Miller et al. 1997).

In another study, Epstein and colleagues (1998) investigated the link between childhood rape, PTSD, and lifetime alcohol use in adult women. The study found that women with a history of childhood rape had twice as many PTSD symptoms as did women without such a history. Furthermore, childhood rape victims had significantly more alcohol symptoms than did non-victims. Finally, childhood rape victims with PTSD symptoms had twice as many alcohol-related symptoms as did victims without PTSD symptoms. These authors suggest that PTSD may be one of the mediators between childhood rape and alcohol use. For example, people experiencing PTSD symptoms might use alcohol to gain relief from the persistent memories of the abuse.

## Conclusion

Researchers have studied alcohol abuse as both a contributor to and a consequence of child abuse. To date, studies have not determined conclusively the role of parental alcohol abuse in the perpetration of physical or sexual child abuse or neglect. However, several studies have indicated that parental alcohol abuse may increase a child’s risk of experiencing physical or sexual abuse, either by a family member or by another person. Furthermore, researchers have proposed several hypotheses regarding the mechanisms through which parental alcohol abuse might contribute to the abuse or neglect of children.

The relationship between childhood victimization and adult alcohol abuse appears somewhat more solid, particularly for women who were victims of child abuse and neglect. Thus, studies using various types of samples found that women who had experienced childhood maltreatment were more likely to have alcohol problems as adults than other women and that women undergoing alcoholism treatment were more likely to have been victims of childhood victimization than other women. Too few studies have investigated the relationship between childhood victimization and adult alcohol use among men to permit firm conclusions, but the evidence so far indicates that child abuse and neglect is not an independent risk factor for subsequent alcohol problems in men. Further research is greatly needed in this area. Additional research also is needed on the factors that mediate or moderate the link between childhood abuse and adult alcohol-use patterns. Researchers have suggested several such factors, such as inadequate coping skills, antisocial behavior, and PTSD, but the exact roles of these factors must be elucidated further.

Once the mechanisms underlying the relationship between child abuse and adult alcohol abuse are better understood, clinicians, social workers, and other interested groups can use that knowledge to intervene with the victims of child abuse and help prevent subsequent alcohol problems in those victims. For example, such efforts could be targeted at abused and neglected females (both adolescents and older women) to reduce their risk of adult alcohol problems. Because alcohol use as a coping mechanism has been identified as a mediator between childhood abuse and neglect and subsequent alcohol problems, such interventions could focus on helping those women develop more positive coping mechanisms.

## Footnotes

1 The study included only parents who met the criteria of an alcohol-use disorder as well as another drug-use disorder, but excluded parents with only alcohol-use disorders.

2 Although both men and women can be victims as well as perpetrators of sexual abuse, most studies in this area focus on women (or girls) who are abused by men.

3 In this study, CSA was defined as (1) any intrafamilial sexual activity before age 18 that was unwanted by the woman or involved a family member at least 5 years older than the woman or (2) any extrafamilial sexual activity that occurred before age 18 and was unwanted or that occurred before age 13 and involved a person at least 5 years older than the woman.

4 Diagnostic and Statistical Manual of Mental Disorders, Third Edition, Revised.
